# Randomized clinical trial studying effects of a personalized supervised lifestyle intervention program on cardiovascular status in physically inactive healthy volunteers

**DOI:** 10.18632/oncotarget.23958

**Published:** 2018-01-03

**Authors:** Helena U Westergren, Li-Ming Gan, Marianne Månsson, Sara Svedlund

**Affiliations:** ^1^ Department of Molecular and Clinical Medicine, Institute of Medicine at Sahlgrenska Academy, University of Gothenburg and Sahlgrenska University Hospital, Gothenburg, Sweden; ^2^ Cardiovascular and Metabolic Diseases, Department of Personalized HealthCare and Biomarkers, AstraZeneca R and D Gothenburg, Mölndal, Sweden; ^3^ Cardiovascular and Metabolic Diseases, Innovative Medicines and Early Development, Department of Early Clinical Development, AstraZeneca R and D Gothenburg, Mölndal, Sweden; ^4^ Department of Urology, Institute of Clinical Sciences at Sahlgrenska Academy, University of Gothenburg, Gothenburg, Sweden; ^5^ The Department of Clinical Physiology, Sahlgrenska University Hospital, Gothenburg, Sweden

**Keywords:** coronary flow reserve, physical exercise, microvascular dysfunction, ultrasound

## Abstract

**Background:**

The impact of personalized exercise training and a healthy dietary lifestyle in healthy volunteers on coronary flow reserve and cardiovascular function remains to be investigated in a controlled study setting.

**Purpose:**

To examine the effects of a Mediterranean-inspired diet combined with regular physical exercise (standard) and a personalized supervised exercise program (DAPS) on coronary flow reserve and cardiovascular function.

**Results:**

The number of males were 10 (59%) and 9 (47%) and mean age was 54 ± 12 and 55 ± 5 years in standard versus DAPS group, respectively. Primary outcomes were in addition to improved body composition and aerobic capacity, increased TDE-CFR (5.0%, CI:1.62,8.64, *p* = 0.005) and left ventricle ejection fraction (LVEF) during hyperemia (10.2%, CI:1.62,19.4, *p* = 0.022) in DAPS adjusted for the control period. Also, plasma fibrinogen decreased (−12.1%, CI:-22.0,–0.92, *p* = 0.035) in the DAPS group. Secondary outcomes, after adjusting DAPS intervention effects for the standard-training period, TDE-CFR and hyperemic LVEF remained significantly improved.

**Materials and Methods:**

This randomized, controlled clinical trial (URL: http://www.clinicaltrials.gov NCT02713724) included 36 healthy volunteers who underwent exercise ECG before randomization to standard or DAPS groups. Standard-group was given gym-membership with limited instructions and general dietary advice. DAPS-group received personalized supervised exercise programs and more detailed dietary advice with regular contact with a personal trainer. Effects were evaluated after 3 months. All participants underwent coronary flow reserve by transthoracic ultrasound (TDE-CFR), blood marker analysis and examinations of vascular function. Standard-group was evaluated pre-control, post-control (=pre-intervention) and post-intervention. DAPS-group was examined at pre-intervention and post-intervention.

**Conclusions:**

A personalized supervised training- and diet program improves cardiovascular status in healthy subjects with a physically inactive lifestyle and may be a promising approach for cardiovascular prevention in the general population.

## INTRODUCTION

From a global perspective, overweight and obesity are established health issues contributing to devastating morbidity and mortality from metabolic and cardiovascular related diseases. The epidemiology of a physically inactive lifestyle is increasing, and the number of young persons and adolescents at risk are overwhelming. Exercise training is well known to have beneficial effects on cardiovascular status [1, 2]. In an interesting observational study, persons who conduct regular low-level physical exercise with only 15 minutes daily, seem to have a longer expected life time and also a longer healthy life expectancy [3].

However, effects of physical activity on cardiovascular health have mostly been evaluated in observational studies, there are a limited number of randomized studies assessing the effects of physical exercise on coronary microvascular function despite its importance for cardiovascular prognosis [4]. A positive effect of six months aerobic exercise on coronary flow reserve (CFR) have been demonstrated in healthy volunteers [5] and was recently shown to be beneficial in patients with coronary artery disease (CAD) already after three months [6]. WHO’s Global Recommendations on Physical Activity for Health [7] as well as 2008 physical activity guidelines for Americans [8] recommends adults at least 150 minutes of moderate-intensity training spread out throughout the week. One fourth of the adult population in the European Union do not meet the recommendations from WHO on physical activity, with large variation between countries [9], while only 50% of the Americans seem to fulfil these guidelines [10]. Furthermore, not only physical exercise but also healthy dietary habits are of importance in cardiovascular prevention [1] and Mediterranean diet is one of the most studied showing beneficial effects on cardiovascular health [11]. Thus, for optimal cardiovascular prevention, an easily accessible and adaptable program including physical exercise and dietary advice may be of great value for improved uptake and adherence, and thereby useful in daily life. In the current study, we aimed to evaluate a health intervention program in a controlled clinical trial, including a combination of personalized physical exercise with moderate intensity and Mediterranean-inspired dietary advice to achieve beneficial effects on cardiovascular status in healthy volunteers with physically inactive lifestyle.

## RESULTS

### Baseline characteristics of participants and study completion

The study population consisted of 36 recruited physically inactive healthy subjects between 38–64 years of age and with a BMI range of 20–26. None of the participants had ongoing or a previous history of cardiovascular disease. Clinical characteristics of the whole study population are presented in Table 1. The number of males was 10 (59%) and 9 (47%) and mean age was 54 ± 12 years and 55 ± 5 years in standard versus DAPS group, respectively. Of the 36 participants, three did not complete the hyperemic echocardiography investigation and TDE-CFR protocol. Two of the participants had incomplete EndoPAT investigations and up to six had missing data on laboratory blood analyses.

**Table 1:** Baseline characteristics of study cohort

	Whole study population (*n* = 36)
Male (number, %)	53 (19)
Age (years)	54 ± 6
Body mass index (kg/m^2^)	24.0 (23.0;25.0)
Waist/Hip ratio (*n* ***=*** 33)	0.85 (0.81;0.89)
Fat percentage (%) (*n* ***=*** 32)	29.7 (27.2;32.9)
SBP (mmHg)	118 (110;125)
DBP (mmHg)	70 (70;80)
Maximal exercise capacity (W)	162 ± 45
LVEF (%)	65 ± 7
Cardiac Output (10^2^)	35 (33;43)
Heart rate (bpm)	61 ± 10
Total cholesterol (mmol/L) (*n* ***=*** 34)	5.4 ± 0.9
Triglycerides (mmol/L) (*n* ***=*** 34)	0.77 (0.58;1.05)
HDL (mmol/L) (*n* ***=*** 34)	1.85 (1.5;2.23)
Fasting Glucose (mmol/L) (*n* ***=*** 34)	5.2 ± 0.4
Insulin (mU/L) (*n* ***=*** 30)	4.6 (3.1;6.5)
HOMA-IR (*n* ***=*** 30)	1.1 (0.7;1.6)

Data are presented with mean ± SD or median ± interquartile range for normally distributed and non-normally distributed, respectively. DBP = diastolic blood pressure; HDL=high density lipoprotein; HOMA-IR = homeostatic model assessment of insulin resistance; LVEF = left ventricle ejection fraction; SBP = systolic blood pressure.

### Effects of DAPS compared to matched and time-aligned controls

#### Effects on body composition and cardiovascular system

The primary objective of the study was to compare the control period of the standard group with age, gender and BMI matched participants in the DAPS group (Figure 2). In the current study, variance of coefficient of TDE-CFR measurement for the control period was 4.0%. Mean change of TDE-CFR during the intervention period with a personalized supervised health program of three months was significantly increased in DAPS (6.1% (CI:3.7,8.5%), *p* < 0.001) but unchanged in the control group (1.5% (CI:-0.9,4.1%), *p* = 0.198) (Figure 3A). Also LVEF was improved in the DAPS group (6.2% (CI:1.9,10.7%), *p* = 0.007) but unaltered in controls (−3.6% (CI:-11.9,5.5%), *p* = 0.381) (Figure 3B). The significant increase of TDE-CFR and LVEF in DAPS remained with 5.0% and 10.2%, respectively when corrected for controls (Table 2). Furthermore, also the decrease in BMI (DAPS: −3.9% (CI:–6.0,–1.7%), *p* = 0.002; Controls: −0.7% (CI:–3.0,1.7%), *p* = 0.560), waist/hip ratio (DAPS: −3.1% (CI:–4.7,−1.4%), *p* = 0.001; Controls: -0.1% (CI:-2.5,2.4%), *p* = 0.938) as well as fat percentage (DAPS: 5.1% (CI:–7.8,−2.4%), *p* = 0.001; Controls: 0.2% (CI:-1.9,2.3%), *p* = 0.861) remained significant in the DAPS group with a decrease of 3.6%, −3.0% and −5.2%, respectively when corrected for controls (Table 3). All analyzed parameters are listed in Tables 2 and 3.

**Figure 1:**
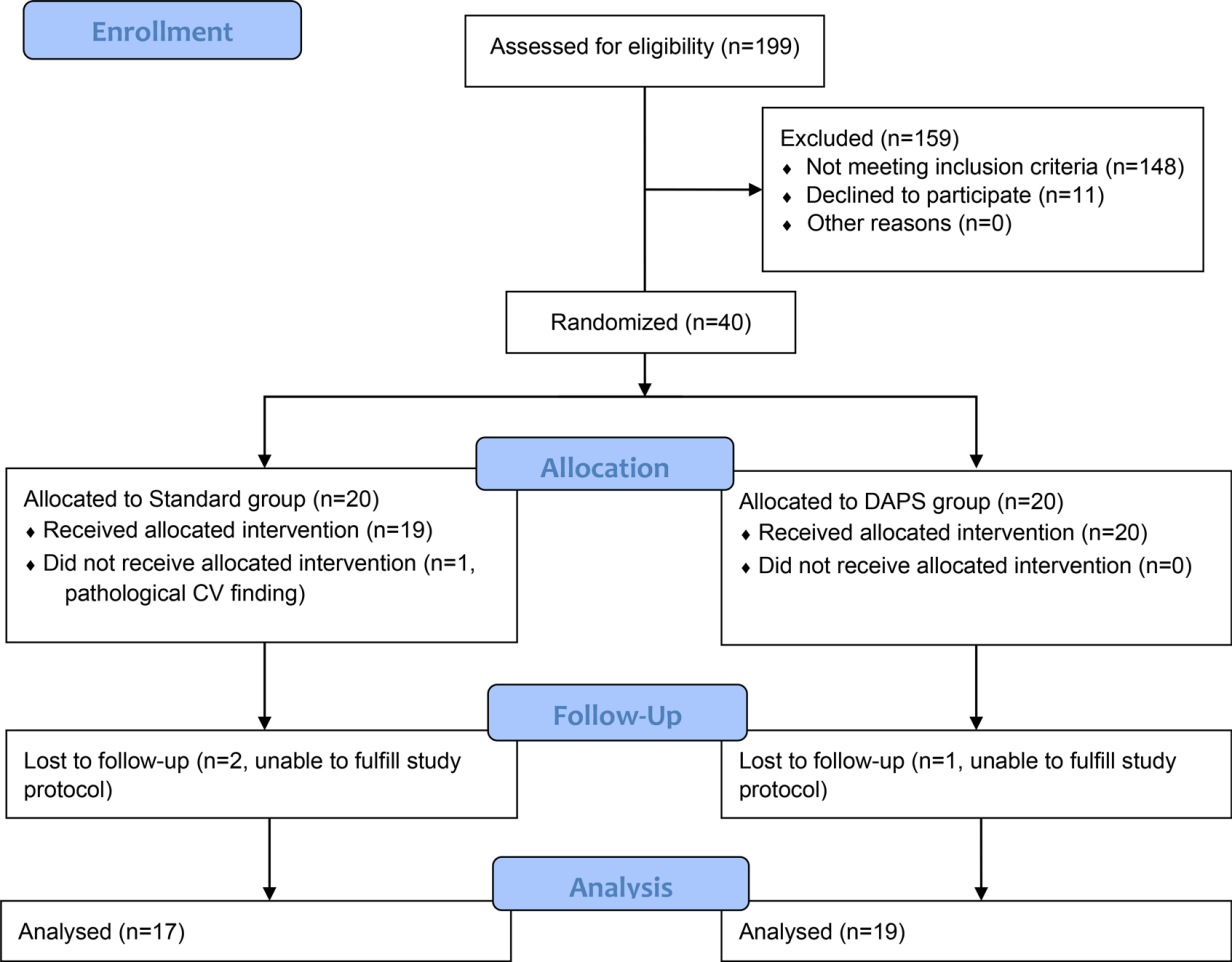
Recruitment and follow-up of study participants CV=cardiovascular; DAPS=Diagnosis, Analysis, Personalization, Supervision.

**Figure 2:**
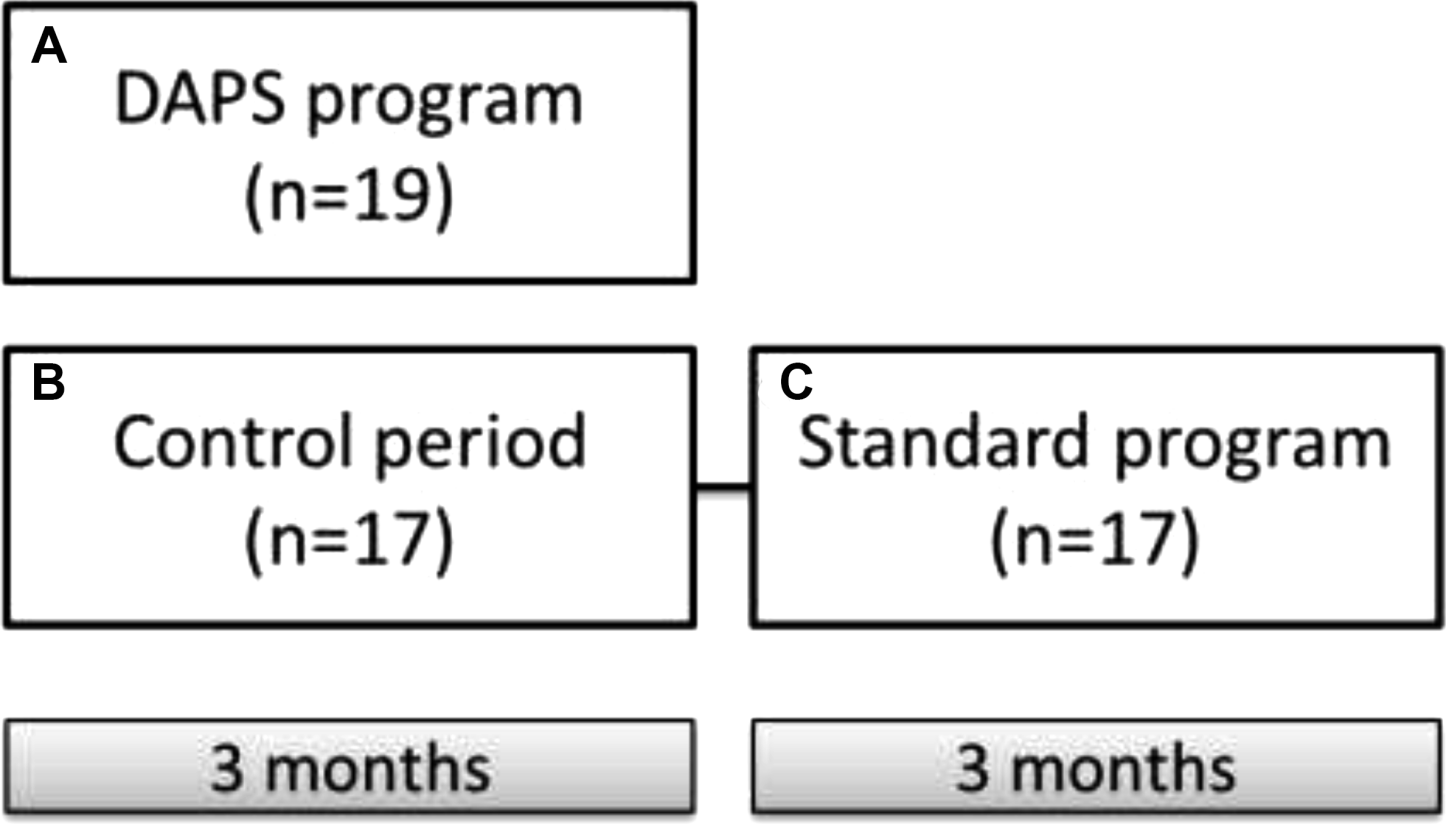
Study design Schematic overview of study design. Subjects were randomized into DAPS with a three months intervention period as well as a standard group including a three months control period followed by a three months standard exercise period. The primary objective of the current study was to compare effects of the DAPS program (**A**) including personalized exercise training and Mediterranean-inspired diet with a time-aligned initial control period of the standard group (**B**). Secondary objective was to compare the DAPS program (A) with the standard program (**C**), mimicking a regular gym and Mediterranean-inspired nutrition program. Finally, the third objective was to compare the standard program (C) with its own control period (B). DAPS = Diagnosis, Analysis, Personalization, Supervision.

**Figure 3:**
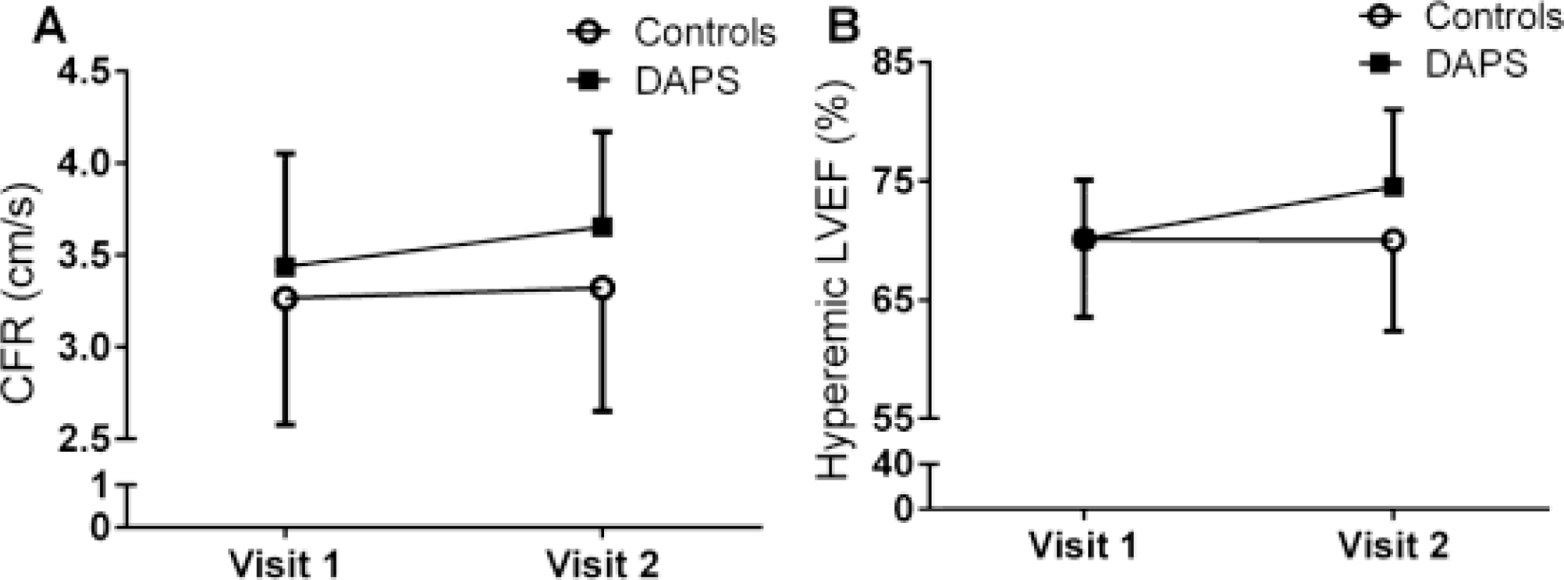
Change in coronary flow reserve and hyperemic left ventricle function after a personalized supervised health intervention program The number of 36 healthy inactive volunteers were recruited for study participation and randomized into a three months DAPS program (*n* = 19) or a three months control period followed by three months standard intervention program (*n* = 17). (**A**) Shows the increase in CFR after DAPS intervention compared to controls, *p* = 0.005. (**B**) Displays the amelioration in hyperemic left ventricle ejection fraction in the DAPS group compared to controls, *p* = 0.022. Data are presented with mean ± SD. CFR = coronary flow reserve; DAPS = Diagnosis, Analysis, Personalization, Supervision; LVEF = Left Ventricle Ejection Fraction.

**Table 2:** Effect of personalized overall health program on physiological testing in DAPS compared to controls using ANCOVA

	DAPS pre-exercise (*n* = 19)	DAPS post-exercise (*n =* 19)	Standard pre-control (*n* = 17)	Standard post-control = pre-exercise (*n* = 17)	Adjusted effect (%) (95% CI)	*p*-value (adjusted effect)
Basal flow (cm/s)	0.21 (0.19;0.23)	0.20 (0.17;0.23)	0.19 (0.17;0.22) (*n* = 14)	0.19 (0.17;0.22) (*n* = 14)	−4.50 (−9.64,0.93)	0.096
Hyperemic flow (cm/s)	0.71 ± 0.12	0.73 ± 0.12	0.62 ± 0.07 (*n* = 14)	0.63 ± 0.08 (*n* = 14)	−0.46 (−5.38,4.95)	0.886
TDE-CFR	3.44 ± 0.61	3.64 ± 0.53	3.28 ± 0.69 (*n* = 14)	3.33 ± 0.68 (*n* = 14)	4.95 (1.62,8.64)	0.005
LVEF rest (%)	65 ± 8	64 ± 13	66 ± 8	65 ± 9	−0.92 (−8.59,7.65)	0.829
Hyperemic LVEF (%)	70 ± 5	75 ± 7	70 ± 7 (*n* = 14)	68 ± 10 (*n* = 14)	10.15 (19.40, 1.62)	0.022
HR rest (bpm)	60 ± 10	55 ± 6	62 ± 12	57 ± 7	−1.83 (−7.53, 4.23)	0.539
HR hyperemia (bpm)	80 ± 16	77 ± 10	84 ± 17 (*n* = 14)	77 ± 14 (*n* = 14)	4.95 (−3.39,14.02)	0.244
CO rest (10^2^)	34 (25;34)	33 (27;36)	36 (33;47)	37 (27;41)	−4.94 (−18.15,10.15)	0.487
Rate pressure product (bpm x mmHg x 10^2^)	73 (61;85)	67 (56;75)	70 (61;81)	67 (62;76)	−4.06 (−10.67,3.28)	0.270
RHI	2.44 (2.09;2.68) (*n* = 18)	2.45 (2.12;3.01) (*n* = 18)	2.41 (2.18;2.91) (*n* = 16)	2.46 (2.12;3.25) (*n* = 16)	2.09 (−12.70,19.40)	0.749
cbIMT (mm)	1.05 (0.90;1.15)	1.10 (1.00;1.20)	1.00 (0.85;1.13)	1.10 (0.85;1.10)	4.47 (−3.39,12.98)	0.268
Carotid IMT (mm)	0.64 (0.59;0.77)	0.62 (0.57;0.72)	0.61 (0.55;0.64)	0.63 (0.54;0.66)	−4.28 (−9.84,1.62)	0.145

Data are presented with mean ± SD or median ± interquartile range for normally distributed and non-normally distributed, respectively. Effect of DAPS adjusted for controls is presented as percentage change with 95% confidence intervals. cbIMT=carotid bifurcation intima-media thickness; CI = confidence interval; CO = cardiac output; HR = heart rate; IMT=intima media thickness; LVEF=left ventricle ejection fraction; RHI = reactive hyperemic index; TDE-CFR = transthoracic color Doppler echocardiography assessed coronary flow reserve.

**Table 3:** Effect of personalized overall health program on clinical parameters and biomarkers in DAPS compared to controls using ANCOVA

	DAPS pre-exercise (*n* = 19)	DAPS post-exercise (*n* = 19)	Standard pre-control (*n* = 17)	Standard post-control = pre-exercise (*n* = 17)	Adjusted effect (%) (95% CI)	*p*-value (adjusted effect)
Body mass index (kg/m^2^)	24.0 (23.0;24.3)	23.0 (21.0;24.0)	24.0 (23.0;25;0)	24.0 (22.5;24.8)	−3.62 (−6.46,−0.69)	0.017
Waist/Hip ratio	0.85 (0.79;0.89) (*n* = 17)	0.82 (0.77;0.89) (*n* = 17)	0.85 (0.81;0.90) (*n* = 16)	0.85 (0.80;0.90) (*n* = 16)	−2.95 (−5.59,−0.09)	0.044
Fat percentage (%)	29.9 (28.2;32.3) (*n* = 17)	28.8 (26.7;30.9) (*n* = 17)	29.6 (25.0;33.1) (*n* = 15)	29.4 (25.7;33.7) (*n* = 15)	−5.16 (−8.59,−1.83)	0.004
hs-CRP (nmoL/L)	3.81 (2.48;11.1) (*n* = 18)	2.95 (0.76;10.9) (*n* = 18)	6.57 (3.24;7.81) (*n* = 16)	4.00 (2.76;7.33) (*n* = 16)	−23.62 (−59.45,43.55)	0.390
Cholesterol (mmol/L)	5.4 ± 0.8 (*n* = 18)	5.3 ± 0.8 (*n* = 18)	5.5 ± 0.9 (*n* = 16)	5.5 ± 0.8 (*n* = 16)	−4.28 (−8.80,0.69)	0.088
Triglycerides (mmol/L)	0.73 (0.56;0.96) (*n* = 18)	0.67 (0.60;0.98) (*n* = 18)	0.86 (0.65;1.25) (*n* = 16)	0.82 (0.61;1.14) (*n* = 16)	3.99 (−9.64,19.40)	0.573
HDL (mmol/L)	1.80 (1.60;2.08) (*n* = 18)	1.80 (1.55;2.20) (*n* = 18)	1.95 (1.43;2.28) (*n* = 16)	1.80 (1.50;2.25) (*n* = 16)	3.04 (−3.84,10.66)	0.385
Total leukocyte count (^*^10^9^/L)	5.70 (4.88;6.60) (*n* = 18)	4.75 (4.90;6.60) (*n* = 18)	5.05 (4.70;6.75) (*n* = 16)	4.80 (4.25;5.60) (*n* = 16)	−4.28 (−17.40,10.92)	0.549
Platelet count (^*^10^9^/L)	219 (203;253) (*n* = 18)	216 (181;229) (*n* = 18)	226 (212;258) (*n* = 16)	227 (212;261) (*n* =16)	−12.30 (−19.65,−4.06)	0.005
Neutrophils (^*^10^9^/L)	2.95 (2.50;3.85) (*n* = 18)	2.60 (1.80;3.30) (*n* = 18)	2.85 (2.18;4.30) (*n* = 16)	2.55 (2.10;3.30) (*n* = 16)	−7.32 (−26.38,16.68)	0.507
Fibrinogen (μmol/L)	9.11 (8.38;11.3) (*n* = 14)	8.23 (7.35;10.7) (*n* = 14)	8.53 (8.23;10.0) (*n* = 16)	8.53 (7.94;10.0) (*n* = 16)	−12.10 (−22.02,−0.92)	0.035
Fasting Glucose (mmol/L)	5.2 ± 0.4 (*n* = 21)	5.2 ± 0.4 (*n* = 21)	5.3 ± 0.4 (*n* = 16)	5.3 ± 0.3 (*n* = 16)	−1.60 (−5.38,2.33)	0.419
Insulin (pmol/L)	32.6 (22.2;50.0) (*n* = 14)	31.3 (25.0;43.8) (*n* = 14)	32.0 (19.5;42.4) (*n* = 16)	29.9 (16.0;39.6) (*n* = 16)	21.62 (−10.05,64.06)	0.193
HOMA-IR	1.1 (0.7;1.9) (*n* = 14)	1.0 (0.8;1.4) (*n* = 14)	1.0 (0.7;1.5) (*n* = 16)	1.0 (0.5;1.4) (*n* = 16)	18.85 (−13.90,64.06)	0.279
Quality of Life (% of max)^*^	80 (72;83)	83 (76;90)	83 (80;87) (*n* = 15)	83 (73;88) (*n* = 15)		0.049

Data are presented with mean ± SD or median ± interquartile range for normally distributed and non-normally distributed, respectively. Effect of DAPS adjusted for controls is presented as percentage change with 95% confidence intervals. CI = confidence interval; HDL = high density lipoprotein; HOMA-IR = homeostatic model assessment of insulin resistance; hs-CRP = high sensitivity C-reactive protein.

^*^Analyzed on delta change using Wilcoxon signed-rank test

### Effects on biomarkers

Among the analyzed blood markers (Table 3), the decrease in fibrinogen (DAPS: 11.1% (CI:–18.4,–3.2%), *p* = 0.011; Controls: 0.5% (CI:–8.0,9.8%), *p* = 0.915) and platelet count (DAPS: 7.8% (CI:-14.2,–1.0%), *p* = 0.028; Controls: 5.2% (CI:–0.6,11.3%), *p* = 0.076) during the personalized supervised health program in the DAPS group remained significant when corrected for controls with 12.1% and -12.3%, respectively (Table 3).

### Effect of DAPS compared to standard program

#### Effects on body composition, cardiovascular system and biomarkers

The second objective of the study was to compare the DAPS group receiving a personalized supervised program with the matched participants performing the standard program (Figure 2). Only the parameters significantly different between DAPS and controls (Table 2 and 3) were analyzed (Table 4). Briefly, TDE-CFR and hyperemic LVEF remained significantly increased with 4.0% and 8.9%, respectively in DAPS after adjusted for the standard program.

**Table 4:** Effect of personalized overall health program on clinical parameters and biomarkers in DAPS compared to a standard program using ANCOVA

	DAPS pre-exercise (*n* = 19)	DAPS post-exercise (*n* = 19)	Standard post-control = pre-exercise (*n* = 17)	Standard post-exercise (*n* = 17)	Adjusted effect (%) (95% CI)	p-value (adjusted effect)
TDE-CFR	3.44 ± 0.61	3.64 ± 0.53	3.33 ± 0.68 (*n* ***=*** 14)	3.40 ± 0.62 (*n* ***=*** 14)	3.99 (0.69,7.65)	0.018
Hyperemic LVEF (%)	70 ± 5	75 ± 7	68 ± 10 (*n* ***=*** 14)	67 ± 7 (*n* ***=*** 14)	8.89 (1.62,16.68)	0.017
Body mass index (kg/m^2^)	24.0 (23.0;24.3)	23.0 (21.0;24.0)	24.0 (22.5;24.8)	23.0 (21.5;24.5)	−1.83 (−4.50,0.93)	0.180
Waist/Hip ratio	0.85 (0.79;0.89) (*n* ***=*** 17)	0.82 (0.77;0.89) (*n****=*** 17)	0.85 (0.80;0.90) (*n* ***=*** 16)	0.80 (0.76;0.87) (*n* ***=*** 16)	1.16 (−2.28,4.95)	0.497
Fat percentage (%)	29.9 (28.2;32.3) (*n* ***=*** 17)	28.8 (26.7;30.9) (*n****=*** 17)	29.4 (25.7;33.7) (*n* ***=*** 15)	29.3 (24.8;32) (*n* ***=*** 15)	−2.05 (−5.59,1.39)	0.222
Platelet count (^*^10^9^/L)	219 (203;253) (*n****=*** 18)	216 (181;229) (*n****=*** 18)	227 (212;261) (*n****=*** 16)	211 (197;256) (*n* ***=*** 16)	−2.50 (−10.05,5.68)	0.540
Fibrinogen (μmol/L)	9.11 (8.38;11.3) (*n* ***=*** 14)	8.23 (7.35;10.7) (*n****=*** 14)	8.53 (7.94;10.0) (*n* ***=*** 16)	8.82 (8.03;9.41) (*n* ***=*** 16)	−6.89 (−16.05,3.28)	0.170
Quality of life (%)^*^	80 (72;83)	83 (76;90)	83 (73;88) (*n* ***=*** 15)	89 (85;91) (*n* ***=*** 15)		0.860

Data are presented with mean ± SD or median ± interquartile range for normally distributed and non-normally distributed, respectively. Effect of DAPS relative to standard program is presented as percentage change with 95% confidence intervals. LVEF = left ventricle ejection fraction; TDE-CFR = transthoracic color Doppler echocardiography assessed coronary flow reserve.

^*^Analyzed on delta change using Wilcoxon signed-rank test

### Effects on physical improvement

The participants estimated to have fulfilled the exercise training by 92% and 79% (*p* = 0.142) and the diet advice by 84% and 79% (*p* = 0.688) in DAPS versus standard, respectively. Furthermore, biceps curls (DAPS: 1.25 ± 1.39, *p* = 0.004; standard: 1.90 ± 0.99, *p* = 0.002) and chest press (DAPS: 1.31 ± 1.20, *p* = 0.002; standard: 1.30 ± 0.82, *p* = 0.004), was improved after both interventions. Aerobic capacity (DAPS: 0.59 ± 0.87, *p* = 0.028; standard: 0.30 ± 0.67, *p* = 0.500) and balance (DAPS: 0.53 ± 0.62, *p* = 0.008; standard: 0.00 ± 1.25, *p* = 1.00) were statistically improved in the DAPS group after intervention, while horizontal jumps (DAPS: 0.29 ± 0.47, *p* = 0.062; standard: 0.30 ± 0.48, *p* = 0.250) remained statistically unaltered in both groups. Of note, the physical improvements tests before and after the exercise period were only completed by 59% of the participants in the standard group and of 89% in the DAPS group.

### Effect of standard program on body composition, cardiovascular system and biomarkers

The third objective of the study was to compare the participants in the standard group with its own control period (Figure 2). The standard intervention did not statistically affect TDE-CFR (2.52% (CI:-0.38,5.34%), *p* = 0.083) nor hyperemic LVEF (−0.83% (CI:-10.34,7.85%, *p* = 0.841). However, a decrease was observed in BMI with -2.11% (CI:-4.08,−0.17%, *p* = 0.035) in estimated fat percentage with -3.40% (CI:-6.13,−0.73%, *p* = 0.016) in waist/hip ratio with 4.67% (CI:-8.57,−0.91%, *p* = 0.018) and in platelet count with -6.80% (CI:-11.56,−2.24%, *p* = 0.006), after intervention. Although, only platelet count remained statistically decreased when comparing to the control period -12.3% (CI:-22.19,−3.25, *p* = 0.010). We observed no further statistical difference in the physiological investigations, clinical parameters or blood markers from the standard program compared to the control period, data not shown.

## DISCUSSION

The current study aimed to investigate the effect of a personalized supervised lifestyle intervention program, including physical exercise and dietary habit improvements, on microvascular function and cardiovascular biomarkers in physically inactive healthy volunteers with normal CFR. The participants received Mediterranean-inspired diet instructions combined with regular physical exercise (standard group) or a personalized supervised exercise program (DAPS group). Our results indicate that after three months of intervention, TDE-CFR together with LVEF during hyperemia were significantly increased in the DAPS group compared to a time-aligned control period in the standard group. Interestingly, plasma fibrinogen decreased in the DAPS group as compared to controls, further supporting the findings of increased TDE-CFR. Secondly, when comparing the DAPS group to the standard group, again TDE-CFR and hyperemic LVEF were significantly increased. However, we observed no statistically significant differences in the other physiological tests when comparing the standard group to its own control period.

### Impact of DAPS intervention on coronary microvascular function, cardiac capacity and biomarkers

The favorable impact of physical activity on cardiovascular health has been long studied and today a physically inactive life style is considered one of the major modifiable risk factors for cardiovascular disease [2]. In the guidelines from the European Society of Cardiology for prevention of cardiovascular disease (version 2012) not only regular physical exercise but also a healthy diet are considered important cornerstones in preventing cardiovascular disease [1]. Mediterranean diet is one of the most studied in cardiovascular prevention, showing beneficial effects on cardiovascular incidence as well as the components of metabolic syndrome [11]. In this study, we show that a personalized supervised three months health improvement program (DAPS) seems to ameliorate coronary microvascular function in healthy volunteers with already high CFR. This increase is statistically significant both compared to a time-aligned control group as well as to a standard three months health improvement program. In accordance, correlation between exercise capacity and CFR has been demonstrated in heart failure patients [12], CAD patients [6], as well as in healthy volunteers [13, 14]. Olsen et.al. recently demonstrated three months aerobic interval training to be associated with increased CFR with comparable amplitude as the effect of a three month low-energy diet, in obese CAD patients [6]. This shows both increased fitness and reduced fatness to be important for coronary microvascular function. The current study indicates a personalized and more intense health intervention (DAPS) to amplify the increase in CFR compared to the standard program. This may involve both increased exercise capacity as indicated by increased cardiac reserve as well as decreased BMI, waist/hip ratio and estimated fat percentage. In agreement, Kiviniemi et.al. showed visceral adipose tissue including waist/hip ratio and adipose secreted peptide hormones (leptin and adiponectin) to be associated with CFR in healthy lean young men [14]. Furthermore, in our study the increase in CFR after DAPS intervention seems due to statistical significantly decreased basal coronary blood flow velocity (*p* = 0.009) before compared to after DAPS intervention. This can at least in part be explained by decreased resting heart rate/rate pressure product and thereby reduced myocardial oxygen demand at rest following intervention, even though these changes were not significantly different in DAPS when adjusted for the control group. Decreased resting coronary blood flow velocity could also be due to increased epicardial coronary blood vessel diameter. Indeed, physical exercise capacity has shown to significantly increase CFR as well as coronary vessel diameter after five months of endurance training in healthy volunteers [5]. Thus, maintained as well as trend towards increased hyperemic flow velocity following training in DAPS, could indicate improved hyperemic volumetric coronary blood flow. Exercise training and weight loss can potentially have favorable both endothelial-independent and dependent functions of the coronary vasculature [15] leading to improved adenosine-induced maximum flow response. Indeed, effect of adenosine is mediated both by metabolic dilatation at a pre-capillary level as well as by flow-mediated nitric oxide-dependent vasodilatation [16]. Exercise training improves endothelial function by increasing nitric oxide production and bioavailability, contributing to a better vasodilator capacity [15]. Taken together, in the current study improved coronary flow velocity reserve could be due to both reduced resting cardiac load as well as improved microvascular function.

Furthermore, CFR is an integrated measure including macro- and microvascular morphology and function. In absence of coronary artery stenosis, vascular function, remodeling, vessel density, systemic inflammation and blood viscosity are determinants of CFR. Blood viscosity is dependent on e.g. lipoprotein and fibrinogen levels [17]. Regular exercise has been demonstrated to lower fibrinogen [18]. In the current study, fibrinogen decreased with 11% in the DAPS group, confirming previous data. In addition, we observed a small but statistical significant decrease in platelet count after both interventions. The physiological effect of this decrease is uncertain; however, our previous study with well-controlled intake of Mediterranean diet has shown to decrease platelet count with 15% in healthy volunteers [19]. Thus, improved rheological environment may also contribute to improve CFR.

Recently vasodilator stress-echo has been recommended by ESC guidelines to diagnose ischemic heart disease [20]. Indeed, adenosine is known to induce peripheral vasodilatation, leading to compensatory heart rate elevation and thereby increased rate pressure product. Hyperemic LVEF was used in the current study to assess cardiac performance/reserve during stress. Improved hyperemic LVEF observed in the DAPS group indicates improved cardiac performance which is in line with the increased CFR.

### Impact of DAPS intervention on carotid arterial wall structure

IMT of the common carotid artery is a marker of structural arterial wall changes known to predict cardiovascular outcome [21]. Self-reported 12 month retrospective exercise training has been shown to be associated with both decreased carotid IMT and carotid vascular stiffness index [13]. In the current study we could not detect any effect of three months combined exercise training and diet-advice on neither carotid IMT nor cbIMT within or between groups. In agreement, three months of endurance training did not alter carotid IMT in healthy middle-aged men [22]. Carotid IMT has been shown to decrease after 8 weeks exercise training in subjects with initial higher carotid IMT [23] as well as in obese men [24]. However, the current population was normal weighted with normal carotid IMT, probably partly explaining the lack of effect. Lakka et al. demonstrated cardio-respiratory fitness to be associated with slower progression of carotid IMT in middle-aged men during a 4-year follow-up [25]. The Meteor study showed the progression of carotid IMT to be about 13μm/year [26]. Thus, the authors believe that in healthy subjects with normal carotid arteries, the current three months interventions may be too short as well as underpowered to detect changes in carotid IMT as well as on cbIMT. However, a shorter period of exercise training has been demonstrated to decrease carotid arterial wall stiffness index (β stiffness index) with 20% in healthy volunteers after three months exercise training [27]. Also physical activity in adolescence is associated with improved carotid artery elasticity later in life [28]. Interestingly, in our study carotid β stiffness index was decreased after DAPS intervention with 19% but unaltered in controls and the standard group (data not shown), confirming previous results. However, the effect was not statistical significant in DAPS when adjusting for controls (data not shown). Nevertheless, even though short-time intervention does not reverse carotid artery structure in this healthy cohort, the trend of improved β stiffness index is also in line with the improved large vessel function.

### Impact of DAPS intervention on peripheral vascular function

RHI is a complex measure of peripheral vascular function, shown to be associated with coronary artery function [29]. Exercise training and calorie restriction for six months increased RHI in obese CAD patients [30], however a recent study did not demonstrate three months of aerobic exercise nor low-energy diet to significantly impact RHI in obese CAD patients [6]. Using RHI as a measure of peripheral vascular function we were not able to detect any changes in the current study. In agreement, in a cross-sectional study increased physical activity was not associated with increased RHI compared to low physical activity in healthy adolescents [31]. However, Shimizu et al demonstrated four weeks blood flow restriction resistance training to improve RHI in healthy elderly subjects with relatively low initial RHI [32]. Also, increased RHI has been associated with decreased Homeostatic model assessment of insulin resistance (HOMA-IR) after 16 weeks of exercise and diet restriction in obese women [33] and high HOMA-IR is related to decreased RHI in patients with suspected myocardial ischemia [34]. Decreased digital vasodilator function derived by EndoPAT has been associated with metabolic risk factors, including obesity, diabetes mellitus, and ratio of total to HDL cholesterol [35]. In our study, the participants were healthy, lean, with normal RHI values as well as unchanged HOMA-IR, HDL and total cholesterol levels after interventions, possibly explaining the lack of effect in this healthy population. Finally, RHI derived with EndoPAT technology in addition to reflect endothelial function is also under influence of digit specific structure and physiology [36], tissue response to ischemia as well as sympathetic nerve inflow [37]. All these confounders could de-mask potential improved peripheral vascular function, as observed at the level of coronary vasculature. Of note, the substantial method variability of 18% in the current study could reduce the chance to detect any potential beneficial effects, despite the operator-independent nature of the method.

### Study limitations

The current study was primarily designed and powered for comparison of DAPS versus controls, therefore secondary and primary objectives comparing DAPS versus standard and control versus standard respectively, should be interpreted with precaution. Furthermore, the power calculation underlying the current study size was performed for CFR, consequently lack of effect on other parameters could be due to poor statistical power and the limited number of subjects. Also, the study was designed to study an easily accessible lifestyle intervention approach combining physical exercise and dietary advice therefore it’s impossible to separate the effects of each intervention per se. The trial was designed to study short duration effects of lifestyle changes and thus, improvements requiring longer intervention have not been assessed. Finally, overweight and/or obese patients are less likely to engage with more intensive exercise programs such as the DAPS intervention and it might therefore be more suitable for lean healthy individuals promoting cardiovascular health.

## MATERIALS AND METHODS

### Study population and design

A total of 40 healthy subjects were recruited for study participation in the current randomized, controlled clinical trial designed to evaluate lifestyle change intervention on cardiovascular function. All subjects were recruited by advertising. The study participants were enrolled at Sahlgrenska University Hospital, Gothenburg starting in year 2015. Study recruitment and management of completion are shown in Figure 1. Inclusion criteria were 1) normal exercise electrocardiogram (ECG) during pre-screening; 2) 35–65 years of age; 3) body mass index (BMI) 20–27; 4) no current or previous history of cardiovascular disease; 5) no family history of cardiovascular disease at an age below 55; 6) no ongoing regular exercise (< 150 min/week) and 7) non-smoker. Exclusion criteria was cardiovascular disease. All participants underwent exercise ECG before study participation. Participants were randomized by a computer software operated by a statistician into two groups, stratified regarding to age, gender and BMI. Both groups were sealed for allocation until start of intervention. The standard group set out with a three months control period during which they were told to continue to live as previously. The control period was followed by a three months intervention period and participants were given membership to a gym with basic instructions on exercise and a Mediterranean-inspired diet (standard program). The exercise was advised to be performed for one hour, three times a week. The Diagnosis, Analysis, Personalization, Supervision (DAPS) group was given a three months personalized supervised exercise program and Mediterranean-inspired diet advice. The exercise program was to be performed for 30 minutes, six times a week and all DAPS-group participants had regular contact with a personal trainer throughout the study. Nutrition advice was given, following Mediterranean-inspired diet advice according to a concept previously described [19]. Briefly, the participants were given menus including olive oil as main fat source, reduced red meat, increased fish consumption, daily fruit and vegetable intake, fiber rich food, and to drink water as main source of liquid. Effects of interventions were evaluated after 3 months. All participants underwent examinations in the following order; endothelial function (peripheral arterial tonometry (PAT)), carotid artery ultrasound scanning, blood sampling for extensive blood marker analysis, transthoracic echocardiography examination and transthoracic Doppler echocardiography assessed coronary flow reserve (TDE-CFR), before and after intervention. All participants were instructed to abstain from caffeine 12 hours and from nicotine (i.e. snuff) 24 hours prior to the examinations. Standard group was evaluated at three different time points; baseline, post control-period and post intervention. DAPS-group was examined at baseline and post intervention (Figure 2). At each visit body fat percentage was estimated using a bioelectrical impedance weight scale (EKS International AB). Standard procedure for manual blood pressure measurements was performed. BMI and waist/hip-ratio was measured.

### Study objectives

Primary objective was to compare effects on cardiovascular-metabolic parameters between the DAPS group and a time-aligned initial control period of the standard group. Secondary objective was to compare the DAPS group with the intervention period of the standard group. Finally, the third objective was to compare the intervention period of the standard group with its own control period (Figure 2). The study was performed in compliance with the Declaration of Helsinki and has been approved by the Regional Ethics Committee of the University of Gothenburg. The trial is registered at clinicaltrials.gov (NCT02713724).

### Quality of life

To measure quality of life and psychological general well‐being we used a generic self‐administered instrument, the Psychological General Well‐Being index (PGWB index). This instrument was developed for providing a self‐reporting instrument that could be used to measure subjective well‐being or distress in healthy individuals [38]. The PGWB index includes 22 items, divided into six clusters: depressed mood, anxiety, positive well‐being, self-control, general health, and vitality. The subscales used to measure these states have three to five items and scores are calculated for each cluster and for an overall PGWB-score.

### Standard lifestyle intervention program

Standard program is a training method that through various pre-designed plans relate to exercise (muscle building, weight reduction/maintaining, improve your self-image, anti-stress/mood and improve overall health), nutrition (weight gaining, weight reduction or weight maintenance) and seeks to improve the living standards of its users. The standard program achieved its design after completing the control period, through a personal questionnaire where the individual choose one of the proposed goals for physical exercise and nutrition. Each participant was given a plan of nutrition (Mediterranean-inspired) and physical activity by an experienced personal trainer for three months depending on his/her goals. With this program we aimed to simulate the service that any citizen can find in a local gym or the service that anyone could get by going to a nutritionist, aiming to simulate the effects of a standard treatment. The participants received accessibility to a local gym during the whole study period with no further personalized training or feedback during the study period. The program was to be conducted at home or at a gym for 60 minutes, three days a week. All study attendants were tested by the same personal trainer for estimation of aerobic capacity (Rockport Fitness Walking Test) [39] number of repetitions with maximal weight in biceps curls and chest press (numbers), length in horizontal jumps (centimeters) and balance (seconds), before and after intervention as a measure of physical improvement. The improvements were scored on an ordinal scale consisting of 3–7 grades. In addition, after study completion the participants subjectively estimated how well they had completed the standard program in terms of exercise training and Mediterranean-inspired diet advice, respectively. The estimation was categorized by the participants to 0–25%, 25–50%, 50–75% and 75–100% for physical training and diet separately.

### Diagnosis, analysis, personalization, supervision (DAPS) personalized supervised intervention program

The DAPS method is a personal training program that is escorted by two points and important differentiators compared to the standard program: the communication with the participant and the personalization of the program. The DAPS-program achieved its design through a personal questionnaire with focus on analyzing the participants exercise and nutrition habits. A weekly program was coordinated by an experienced personal trainer based on personalized exercise, Mediterranean-inspired diet based on a previous study, food advice adapted to each subject, continuous contact by email and feedback for development of a new weekly program. The personalized program was to be easily accessible and conducted at home for 30 minutes, six days a week using weekly received links to exercise programs shared on social media. All DAPS attendants were tested by the same personal trainer for physical improvements as in the standard group, written above. In addition, after study completion the participants estimated how well they had completed the DAPS program in terms of exercise training and Mediterranean diet-inspired advice, respectively, as written above.

### Exercise ECG

A maximal exercise ECG was performed at study inclusion to screen for obvious signs and symptoms of myocardial ischemia. The examination was performed according to a standard protocol with continuous load elevation. Start load was determined from age, gender and weight-reference material [40] and with aim for a 6–8 minutes test time. Blood pressure and Borg Scale was assessed every other minute.

### Transthoracic echocardiography and coronary flow reserve measurement

A basic transthoracic echocardiography examination was performed by an experienced physician according to current recommendations [41], using Sequoia C256 (Acuson Siemens Medical Solutions USA, Inc. Mountain View, CA 94043 USA) with a 4MHz probe (Acuson 4V1c). Left anterior descending coronary artery (LAD) was visualized using color Doppler. Non-invasive TDE-CFR was assessed in the distal (modified apical two-chamber view) or alternatively mid- distal part of LAD (modified short-axis view) at the anterior interventricular sulcus. The flow velocity profile was registered using pulsed wave Doppler, both at rest and during maximum 5 minutes adenosine-induced hyperemia (140 μg/kg/min). During the procedure, ECG and blood pressure were recorded each minute. For repeated measures, care was taken to perform measurements in the same segment of LAD with the same Doppler angle as the first examination. All images were stored and mean diastolic flow velocity profiles were analyzed off-line by an experienced physician blinded to all other results, using the ultrasound software Image Arena (Tomtec, Unterschleißheim, Germany). CFR was calculated as the ratio between mean diastolic blood flow velocity during hyperemia and rest. Left ventricle ejection fractions were estimated by Simpsons biplane method using apical 4- and 2-chamber views, both during rest and during adenosine-induced hyperemia [13].

### Carotid artery ultrasound

Carotid ultrasound scanning was measured by an experienced sonographer according to the recommendations in “Mannheim Carotid Intima Media Thickness consensus update (2004–2006–2011) [42], based on a standardized protocol for measurement of carotid wall structure. The Acuson SC2000^TM^ ultrasound system was used (Acuson Siemens Medical Solutions USA, Inc. Mountain View, CA 94043 USA) with an 8 MHz transducer (Acuson S2000 9L4). B-mode real-time ultrasound was used and CINE-looped images were stored for offline analysis. Common carotid artery intima media thickness (IMT) as well as carotid bifurcation intima media thickness (cbIMT) was averaged from the left and right arteries.

### Peripheral vascular function assessment by peripheral arterial tonometry

Peripheral vascular function was assessed after an overnight fast by PAT using pneumatic finger-cuffs (Endo-PAT2000, Itamar Medical, Caesarea, Israel). The designed probes measure arterial pulsatile volume changes during 5 minutes’ baseline measurements. Forearm occlusion was ensured for 5 minutes in the non-dominant arm and hyperemic response was registered for 5 minutes post cuff deflation. Reactive hyperemic index is calculated automatically as the ratio between the generated mean pulse wave amplitude signals in specified intervals within the 5 minutes post occlusion and baseline periods, in relationship to the response in the contralateral arm. To account for systemic vascular changes, multiplication with a baseline correction factor (0.2276 × ln (occluded arm’s baseline mean pulse wave amplitude)-0.2) is performed by the system [43].

### Laboratory analyses

Blood sampling was drawn in the morning after an over-night fasting. Serum triglycerides, total cholesterol, high density lipoprotein (HDL) cholesterol, total leukocyte count, neutrophils and total platelet count were analyzed using standard methods. Plasma glucose was measured using a photometric method, serum insulin by immunoassay using ElectroChemiLuminescence technology and plasma fibrinogen by the Clauss clotting method using the STA-R Evolution system. All analyses were performed at the department of Clinical Chemistry, Sahlgrenska University Hospital, Gothenburg Sweden.

### Statistical analyses

Deviations in sample size for the various statistical analyses were due to differences in the availability of clinical demographic data, as well as missing values in some analyzed biomarkers/parameters. Statistical analysis was performed using the statistical package for the social sciences (SPSS) for Windows software (version 21.0, Chicago Inc, USA). The sample size was calculated based on a 10% improvement in TDE-CFR during DAPS intervention, with an alpha level 5% and 80% power. A *p*-value < 0.05 was considered statistically significant. Test of skewness was used to assess normal distribution for the numerical variables, and significantly non-normally distributed variables are expressed as median and inter quartile range, while the others are expressed as mean ± SD. Categorical variables are expressed in frequencies and percentages. When appropriate, data were log-transformed before analysis. Coefficient of variation was calculated as SD(x−y)/mean(x,y) × 100%. Within group comparisons were made by means of linear regression with change from baseline as dependent variables and baseline values as covariates.

First objective; effects of the three months intervention on cardiovascular-metabolic variables in the DAPS-group compared to the control period for the standard group, were analyzed by means of ANCOVA (analysis of covariance). We compared the variables at visit 2, using the values at visit 1 as covariates. Second objective; effects of three months intervention in the DAPS-group compared to the intervention period of the standard group, were analyzed using ANCOVA as described above. The third objective; effects of three months intervention in the standard group compared to its own control period were analyzed using paired *t*-tests on delta control-data versus delta intervention-data.

Ordinal data from test of physical improvements and for quality of life were analyzed using Wilcoxon signed-rank test. Physical improvements are expressed as delta change in mean ± SD. The comparisons are within groups. Also the fulfillments of the exercise and diet advice were analyzed by means of Wilcoxon signed-rank test.

## CONCLUSIONS

A personalized supervised training program including a personal trainer with continuous feedback together with a Mediterranean-inspired diet seems to provide more beneficial effects on cardiovascular function in physically inactive healthy subjects compared to controls. Finally, the present study indicates that it is possible to further improve cardiovascular performance in healthy volunteers by an easily adaptable supervised personalized physical exercise and dietary habit intervention, which may have implication for the use of personalized programs and regular coaching for cardiovascular prevention.

## Abbreviations

BMI: Body mass index
cbIMT: Carotid bifurcation intima-media thickness
CAD: Coronary artery disease
CFR: Coronary flow reserve
DAPS: Diagnosis, Analysis, Personalization, Supervision
ECG: Electrocardiogram
HOMA-IR: Homeostatic model assessment of insulin resistance
IMT: Intima media thickness
LAD: Left anterior descending coronary artery
LVEF: Left ventricle ejection fraction
PAT: Reactive hyperemia index
RHI: Peripheral arterial tonometry
TDE-CFR: Transthoracic Doppler echocardiography-coronary flow reserve
